# Higher Self-Esteem Associated With Less Symptoms of Anxiety and Depression Among Young Adults After the Loss of a Parent to Cancer—A Longitudinal Study

**DOI:** 10.1177/08258597211044585

**Published:** 2021-10-21

**Authors:** Tina Lundberg, Kristofer Årestedt, Ulla Forinder, Mariann Olsson, Carl Johan Fürst, Anette Alvariza

**Affiliations:** 1 Care Sciences and Society, Karolinska Institutet, Huddinge, Sweden; 2 Ersta Sköndal Bräcke University College, Stockholm, Sweden; 3 Karolinska University Hospital, Stockholm, Sweden; 4 Linnaeus University, Kalmar, Sweden; 5 Region Kalmar County, Kalmar, Sweden; 6 Gävle University, Gävle, Sweden; 7 Stockholms Sjukhem Foundation, Stockholm, Sweden; 8 Lund University, Lund, Sweden; 9 Dalen Hospital, Stockholm, Sweden

**Keywords:** anxiety, bereavement, cancer, depression, self-esteem, young adult

## Abstract

**Objective:** The purpose of the study was to examine associations between self-esteem and symptoms of anxiety and depression among young adults who lost a parent to cancer. **Methods:** Older adolescents and young adults, aged 16 to 28 years, who had lost their parent to cancer and had accepted an invitation to join a support group, completed a questionnaire 5 to 8 months after the loss and a similar questionnaire about 10 months later (follow-up). Of a total of 77 young adults who participated in the study, 56 completed both questionnaires. Self-esteem was measured with the Rosenberg Self-Esteem Scale. Symptoms of anxiety and depression were measured with the Hospital Anxiety and Depression Scale. Univariate and multiple linear regression models were used to analyze the associations. **Result:** Self-esteem was significantly associated with symptoms of anxiety and depression at baseline and at follow-up. **Conclusion:** This study reveals that self-esteem is a valuable explanatory variable, and that it is associated with both symptoms of anxiety and depression in bereavement. This new knowledge could be used to guide future support to parentally bereaved young adults.

## Introduction

Losing a parent to cancer is an overwhelming experience in a young person's life. The transition from childhood to adulthood constitutes a time of considerable change involving several areas of life,^1^ which might put older adolescents and young adults (hereafter referred to as young adults) who lose a parent in a particularly vulnerable situation. The recognition of young adults as a group with specific needs is quite recent^2^ and studies focusing on parentally bereaved young adults are scarce.^3^ However, some of the existing research reports symptoms of anxiety and depression among young adults who lost someone close to them.^4^ It has been shown that young adults who lost a parent also are more likely to show symptoms of anxiety and depression compared to nonbereaved.^5^ In a recent study, almost 75% of the parentally bereaved young adults who participated reported symptoms of anxiety, and almost 25% of them reported symptoms of depression; on average 5 to 8 months after the loss.^6^ It is also well known that symptoms of anxiety and depression are a common bereavement outcome among adults who lost a family member to cancer.^7,8^ For example, a 30% prevalence of moderate-to-severe symptoms of anxiety, and almost a 25% prevalence of moderate-to-severe prevalence of symptoms of depression, was reported among adult family members 6 months after the loss.^9^ In a nationwide study involving more than 600 young adults who lost a parent to cancer, half of them reported unresolved grief several years after the loss, which was clearly associated with an increased risk of depression.^10^ However, it is also of importance to recognize that a systematic review of psychiatric symptoms among bereaved youth and young adults has found similar levels of anxiety and depression compared with nonbereaved norms.^11^

Self-esteem, that is, how a person feels about him- or herself regarding their worth and self-acceptance,^12^ has been demonstrated as being of importance among children and adolescents after the loss of a loved one, especially for symptoms of anxiety and depression.^13,14^ Parents play an important role in maintaining and developing positive self-esteem among young persons^15^ and it could be that their self-esteem might be compromised if a supporting parent is lost.^13^ Self-esteem seems to increase the most between the ages of 15 and 30.^12^ However, it is also argued to be a personality trait that endures later in life.^16^ While symptoms of anxiety and depression, as described above, commonly increase after a loss, self-esteem levels were normal about 6 months after the loss of a parent to cancer,^7^ and thus may also be more stable when newly bereaved. No study has been identified by the authors that examine the relationship between self-esteem and anxiety and depression among young adults who have lost a parent due to cancer. Therefore, the overall aim of the present study was to examine the relationship between self-esteem and symptoms of anxiety and depression among young adults who lost a parent to cancer. We tested this by posing 3 research questions: (Q1) “Is self-esteem associated with symptoms of anxiety and depression?”; (Q2) “Is self-esteem associated with future symptoms of anxiety and depression?”; and (Q3) “Is self-esteem associated with changes in symptoms of anxiety and depression over time?”

## Method

### Design

This study used data from a prospective longitudinal study examining the psychosocial well-being after the loss of a parent to cancer among young adults who participated in a support group.

### Sample and Procedure

The sample consisted of young adults, aged 16 to 28 years, who had lost their parent to cancer at least 2 months earlier. Recruitment took place at 3 palliative care services in Sweden where the young adults were about to attend a support group. The support groups were directed at young adults who had lost a parent and the groups met 7 or 10 times, every week or every second week. Each group was led by 2 group leaders with experience of working with bereaved families and who were regularly mentored by a psychotherapist. The purpose of the support groups was to enable sharing and support in grief and there was no specific focus placed on self-esteem. Topics concerning the death itself, new life circumstances, experiences of grief and existential questions, what elements are considered to be supportive, remembering the parent, and the future^17,18^ were discussed in a caring environment.

Eligible study participants were identified and recruited by the group leaders. Before the support groups started, the group leaders met with each of the potential participants to inform them about the support groups as well as about the present study. Those who wanted to participate in the study gave their consent by completing a baseline questionnaire in connection with the first group meeting, which was held, on average, 5 to 8 months after the parent had died. A long-term follow-up assessment was completed, on average, 14 to 18 months after the death. All completed questionnaires were sent directly to the researchers by the participants. Reminders were sent after 3 and 6 weeks if questionnaires were missing. In all, 77 young adults participated in the study, and 56 young adults completed both questionnaires.

Data collection was ongoing from October 2011 through July 2016.

### Ethical Considerations

The authors have considered the risk that the questionnaire may be experienced as strenuous and initiate difficult feelings, therefore the voluntariness has been particularly emphasized. The study follows the Declaration of Helsinki. Approval was granted from The Regional Ethical Review Board in Stockholm, Sweden (No. 2011/419-31/5).

### The Questionnaire

The questionnaire included single items and validated self-reported instruments. The content of the questionnaire was guided by the integrative risk factor framework for the prediction of bereavement outcome,^19^ derived from Cognitive Stress Theory^20^ and the Dual Process Model of Coping with Bereavement.^21^ This framework emphasizes the importance of the interaction between factors such as the nature of the stressor, that is, loss- or restoration-oriented factors,^21^ inter- and intra-personal resources, appraisal and coping processes, and outcomes for predicting bereavement outcome. For the present study, a selection of relevant variables was used.

### Characteristics of the Participants and the Loss

The characteristics of the participants consisted of sociodemographic data, such as age, gender, living condition, partner status, and working condition. Characteristics related to the loss included time since death, gender of a deceased parent, living condition at the time of death, and the point when they became aware of the impending death.

### The Rosenberg Self-Esteem Scale (RSE)

The RSE comprises 10 items rated on a four-point Likert scale ranging from 0 (strongly disagree) to 3 (strongly agree). The item responses are summed into a total score ranging between 0 and 30. A higher score indicates a higher level of self-esteem. Five items must be reversed before summarizing the total score.^22^ RSE is widely used and has shown satisfactory measurement properties,^23^ including for the Swedish translation of the tool.^24^ Cronbach's alpha was 0.87 in the present study, illustrating satisfactory internal consistency.

### The Hospital Anxiety and Depression Scale (HADS)

The HADS consists of 14 items equally divided into 2 subscales measuring symptoms of anxiety and depression. Each item has 4 response options ranging from 0 to 3. Eight items must be reversed before summarizing the subscales. The total score for each subscale ranges from 0 (no symptoms) to 21 (highest level of symptoms).^25^ HADS has been widely used in different samples and has shown satisfactory measurement properties,^26^ including in the Swedish version.^27^ In the present study, internal consistency, measured by Cronbach's alpha, was satisfactory for symptoms of anxiety (0.79) as well as for symptoms of depression (0.73).

### Statistical Analysis

Descriptive statistics were used to present characteristics of the study sample and the loss at baseline and at follow-up. Person means imputation was used to replace missing data that did not exceed 20% for each scale.^28^ For RSE, one missing value was replaced, and for HADS, 2 missing values were replaced.

To examine the associations between self-esteem and symptoms of anxiety and depression (Q1), linear regression analyses in 2 blocks were conducted. In Block I, HADS anxiety and HADS depression at baseline were used as the outcome variables, and self-esteem at baseline was used as the explanatory variable. In Block II, age and gender were entered as adjusting covariates. To examine whether self-esteem was associated with future symptoms of anxiety and depression (Q2), the regression analyses were repeated, but with HADS anxiety and depression from the follow-up assessment as outcome variables. To examine whether self-esteem was associated with changes in symptoms of anxiety and depression over time (Q3), the regressor variable method was used, as we found a relatively strong correlation between the baseline and follow-up assessments of symptoms of anxiety (*r*_s_ = .52) and depression (*r*_s_ = .60).^29^ In Block I, HADS anxiety and HADS depression from the follow-up assessment were used as outcome variables and self-esteem, HADS anxiety and HADS depression from the baseline assessment as explanatory variables. In Block II, age and gender were entered as adjusting covariates. Using the criteria of variance of inflation factor (VIF) <2, no problem with multicollinearity for the explanatory variables was detected in any of the regression models.

The statistical significance level was set at *P* < .05. All statistical analyses were performed using SPSS Statistics version 22 (IBM Corp.).

## Results

### Characteristics of the Participants and the Loss

The median age of the 77 young adults who participated in the study was 24 (range = 16-28) years and more than half (*n* = 44, 58%) had lost their mother. Over a third (*n* = 28, 37%) of these young adults had been aware of the impending deaths for less than a few days before. Of the 56 young adults who completed both questionnaires, the median age was 24 (range: 17-28) years and 32 (57%) had lost their mother. Of these 56 young adults, 19 (35%) had been aware of the impending deaths for less than a few days before (Table 1).

**Table 1.** Characteristics of the Participants and the Loss at Baseline and Follow-Up.

	Valid *n*	Baseline *n* = 77	Valid *n*	Follow-up *n* = 56
Age, Mdn (q1-q3) [range]	76	24 (20-25) [16-28]	56	24 (20-25) [17-28]
Gender, *n* (%)	77		56	
Female		64 (83)		46 (82)
Male		13 (17)		10 (18)
Living conditions, *n* (%)	77		56	
Living alone		22 (29)		19 (34)
Living with a partner		26 (34)		15 (27)
Living with parent		25 (32)		20 (36)
Living with sibling		3 (4)		2 (4)
Living with friend		1 (1)		0 (0)
Time since death, *n* (%)	76		56	
2-4 months		21 (28)		18 (32)
5-8 months		37 (49)		24 (43)
>8 months		18 (24)		14 (25)
Deceased parent, *n* (%)	76		56	
Mother		44 (58)		32 (57)
Father		32 (42)		24 (43)
Lived with the now deceased parent, *n* (%)	75		55	
Yes		43 (57)		31 (56)
Have, but moved before the loss		32 (43)		24 (44)
Has a partner, *n* (%)	76		56	
No		33 (43)		28 (50)
Yes		43 (57)		28 (50)
Working conditions, *n* (%)	77		56	
Working		35 (46)		24 (43)
Studying		28 (36)		22 (39)
Working and studying		7 (9)		6 (11)
Unemployed		5 (7)		2 (4)
Sick leave		1 (1)		1 (2)
Parental leave		1 (1)		1 (2)
Awareness of impending death, *n* (%)	75		55	
Short (no awareness to a few days)		28 (37)		19 (35)
Long (a few weeks or longer)		47 (63)		36 (65)

### Associations Between Self-Esteem and Symptoms of Anxiety and Depression (Q1)

Self-esteem at baseline was significantly associated with symptoms of anxiety and depression at baseline. Higher ratings of self-esteem were associated with less symptoms of anxiety in Block I (*B* = −0.30, *P* < .001), explaining 18% of the total variance in the outcome variable. This association remained (*B* = −0.28, *P* *<* .001) after the model in Block II was adjusted for age and gender. The multiple linear regression model in Block II explained 21% of the total variance in symptoms of anxiety (Table 2).

**Table 2.** Associations Between Self-Esteem and Symptoms of Anxiety and Depression at Baseline.

Outcome variables	Explanatory variables	*n*	Block I			Block II		
			*B*	95% CI	*P*-value	*B*	95% CI	*P*-value
Anxiety	Self-esteem	74	−0.30	−0.45/−0.15	<.001	−0.28	−0.43/−0.13	<.001
	Age					0.12	−0.14/0.38	.353
	Young adult being male					−1.00	−3.24/1.23	.372
	Model statistics		*F*(1, 72) = 15.9, *P* < .001, *R*^2^ = .18			*F*(3, 70) = 6.1, *P* = .001, *R*^2^ = .21		

Depression	Self-esteem	72	−0.25	−0.37/−0.14	<.001	−0.26	−0.37/−0.15	<.001
	Age					0.04	−0.16/0.24	.713
	Young adult being male					1.13	−0.55/2.81	.185
	Model statistics		*F*(1, 70) = 20.23, *P* < .001, *R*^2^ = .22			*F*(3, 68) = 7.32, *P* < .001, *R*^2^ = .24		

Block I—Self-esteem at baseline and anxiety and depression at baseline, un-adjusted model.

Block II—Self-esteem at baseline and anxiety and depression at baseline, adjusted model.

Higher ratings of self-esteem were also associated with less symptoms of depression (*B* = −0.25, *P* < .001), explaining 22% of the total variance in this outcome variable. This association remained (*B* = −0.26, *P* < .001) after the model was adjusted for the covariates included in Block II. None of the covariates showed significant associations with symptoms of depression. The adjusted regression model in Block II explained 24% of the total variance in symptoms of depression (Table 2).

### Associations Between Self-Esteem and Future Symptoms of Anxiety and Depression (Q2)

Self-esteem at baseline was significantly associated with symptoms of anxiety and depression at follow-up. Higher ratings of self-esteem at baseline were associated with less symptoms of anxiety at follow-up in Block I (*B* = −0.29, *P* = .001), explaining 19% of the total variance in the outcome variable. This association remained (*B* = −0.29, *P* = .001) after the model in Block II was adjusted for age and gender. The adjusted linear regression model in Block II explained 22% of the total variance in symptoms of anxiety at follow-up (Table 3).

**Table 3.** Associations Between Self-Esteem at Baseline and Future Symptoms of Anxiety and Depression.

Outcome variables	Explanatory variables	*n*	Block I			Block II		
			*B*	95% CI	*P*-value	*B*	95% CI	*P*-value
Anxiety	Self-esteem	56	−0.29	−0.46/−0.13	.001	−0.29	−0.45/−0.12	.001
	Age					0.19	−0.10/0.47	.192
	Young adult being male					0.23	−2.10/2.56	.844
	Model statistics:		*F*(1, 54) = 12.77, *P* = .001, *R*^2^ = .19			F(3,52) = 4.83, *P* = .005, *R*^2^ = .22		
								
Depression	Self-esteem	56	−0.22	−0.37 / −0.07	.006	−0.23	−0.38/−0.08	.003
	Age					0.21	−0.04/0.45	.101
	Young adult being male					2.73	0.69/4.77	.010
	Model statistics		*F*(1, 54) = 8.20, *P* = .006, *R*^2^ = .13			*F*(3,52) = 5.77, *P* = .002, *R*^2^ = .25		

Block I—Self-esteem at baseline and anxiety and depression at follow-up, un-adjusted model.

Block II—Self-esteem at baseline and anxiety and depression at follow-up, adjusted model.

Higher ratings of self-esteem at baseline were also associated with less symptoms of depression at follow-up (*B* = −0.22, *P* = .006), explaining 13% of the total variance in the outcome variable. This association remained (*B* = −0.23, *P* = .003) after the model was adjusted for the covariates included in Block II. The adjusted linear regression model in Block II explained 25% of the total variance in symptoms of depression. Among the covariates, being male was significantly associated with more symptoms of depression (*B* = 2.73, *P* = .010) (Table 3).

### Associations Between Self-Esteem at Baseline and Changes in Symptoms of Anxiety and Depression Over Time (Q3)

Self-esteem at baseline was significantly associated with changes in symptoms of anxiety over time but not with symptoms of depression. Higher ratings of self-esteem at baseline were associated with decreased levels of anxiety over time in Block I (*B* = −0.17, *P* = .044), explaining 32% of the total variance in the outcome variable. This association remains in the adjusted linear regression model in Block II (*B* = −0.18, *P* = .045) (Table 4).

**Table 4.** Associations Between Self-Esteem at Baseline and Changes in Anxiety and Depression Over Time.

Outcome variables	Explanatory variables	*n*	Block I			Block II		
			*B*	95% CI	*P*-value	*B*	95% CI	*P*-value
Anxiety	Self-esteem	56	−0.17	−0.34/−0.00	.044	−0.18	−0.35/−0.04	.045
	Anxiety baseline		0.40	0.15/0.66	.002	0.39	0.12/0.66	.006
	Age					0.06	−0.22/0.34	.671
	Young adult being male					0.18	−2.01/2.36	.872
								
	Model statistics:		*F*(2, 53) = 12.70, *P* < .001, *R*^2^ = .32			*F*(4, 51) = 6.18, *P* *<* .001, *R*^2^ = .33		
Depression	Self-esteem	54	−0.09	−0.24/0.06	.235	−0.11	−0.26/0.04	.137
	Depression baseline		0.56	0.29/0.82	<.001	0.49	0.23/0.76	.001
	Age					0.13	−0.10/0.35	.271
	Young adult being male					1.85	0.02/3.73	.052
								
	Model statistics		*F*(2, 51) = 15.39, *P* < .001, *R*^2^ = .38			*F*(4,49) = 9.11, *P* < .001, *R*^2^ = .43		

Block I—Self-esteem at baseline and anxiety and depression at follow-up, un-adjusted model.

Block II—Self-esteem at baseline and anxiety and depression at follow-up, adjusted model.

## Discussion

This is one of the first studies examining the association between self-esteem and symptoms of anxiety and depression in young adults who have lost a parent to cancer. The results showed that self-esteem is associated with both symptoms of anxiety and depression, even after controlling for age and gender that could be important covariates of potential importance.

The associations found between self-esteem and symptoms of anxiety and depression build on previous research that has shown that self-esteem strongly predicts symptoms of anxiety and depression among adult caregivers in palliative care, even before the loss.^30^ A meta-analysis of longitudinal studies also found self-esteem to be associated with symptoms of anxiety and depression, however, the association was strongest between self-esteem and depression.^31^ Furthermore, the results of the present study are consistent with previous findings among parentally bereaved young persons (7-21 years), where lower self-esteem, measured at 9 months after a loss, was associated with depression 1 year later. In addition, among those reporting depression in the first 9 months, a depressive episode was more likely to occur within the following year.^32^ Furthermore, the results of the present study are comparable to correlations made between self-esteem and symptoms of anxiety and depression among nonbereaved 15 years old, where self-esteem at baseline showed correlations with symptoms of anxiety and depression at baseline as well as at a 2-year follow-up.^33^ These similarities could suggest that the associations found in the present study are not exclusive to bereaved young adults. It may be that the bereaved young adults in the present study only follow the same pattern of associations between self-esteem and symptoms of anxiety and depression as populations of nonbereaved young adults. It could be that the associations rather are explained by the instability and life changes in this age group instead of the bereavement. Furthermore, it could be that young adult who did not attend support groups would have responded differently. The participants had agreed to participate in a support group. Support group participation could have been declined by those who are coping well, as well as those who were not coping in their bereavement, and a lack of information on nonparticipants prevents further analyses of this aspect. However, the findings of who attends or who does not attend support groups are inconsistent.^34,35^ Further research is needed to examine the associations between self-esteem and symptoms of anxiety and depression to be able to conclude what is specific to bereaved young adults.

### Study Limitations and Strengths

According to the integrative risk factor framework for the prediction of bereavement outcome,^19^ a multitude of factors interact and affect the bereavement process. By using the framework, consideration of the importance of the interaction between factors is ensured. This procedure has strengthened the study; despite including controls for age and gender, associations between the variables under study have been found. All participants had joined a support group; such support is an interpersonal resource within the framework, however, this could not be controlled for due to the lack of nonsupported young adults included in the study. All respondents had participated in support groups and we cannot know how this may have affected their self-esteem and symptoms of anxiety and depression.

Furthermore, it is important to consider selection bias when drawing conclusions for the study. As described in the discussion we do not know if the method of selection, young adults who had agreed to participate in a support group, have biased the result.

Finally, although this study was based on a small sample, associations were still found. Although this study cannot draw any strong conclusions about the causal effects, it is likely that self-esteem may affect symptoms of anxiety and depression due to the stable characteristics of self-esteem compared to symptoms of anxiety and depression. The study adopted a prospective design, where questionnaires were gathered on several occasions. As such, it is unique, as this is a sparsely studied group and thus the voice of parentally bereaved young adults has barely been heard.

### Clinical Implications

The study adds valuable knowledge about the associations between self-esteem and its importance on outcomes such as anxiety and depression, which are well-known outcomes in bereavement, in a group that is overlooked in research. The result is useful for informing professionals who encounter these young adults about the risk of low self-esteem contributing to symptoms of anxiety and depression and it draws attention to the importance of recognizing and offering support to these young adults with low self-esteem.

## Conclusion

This study shows associations between self-esteem and symptoms of anxiety and depression among young adults who lost a parent to cancer. The results further demonstrate associations between self-esteem and future symptoms of anxiety and depression. The results add valuable knowledge about the well-being of parentally bereaved young adults and the importance of self-esteem in symptoms of anxiety and depression in bereavement. This new knowledge on young adults’ psychosocial well-being, can guide supportive interventions targeting bereaved young adults.
